# Effect of 1918 PB1-F2 Expression on Influenza A Virus Infection Kinetics

**DOI:** 10.1371/journal.pcbi.1001081

**Published:** 2011-02-17

**Authors:** Amber M. Smith, Frederick R. Adler, Julie L. McAuley, Ryan N. Gutenkunst, Ruy M. Ribeiro, Jonathan A. McCullers, Alan S. Perelson

**Affiliations:** 1 Theoretical Biology and Biophysics, Los Alamos National Laboratory, Los Alamos, New Mexico, United States of America; 2 Departments of Mathematics and Biology, University of Utah, Salt Lake City, Utah, United States of America; 3 Department of Immunology and Microbiology, University of Melbourne, Victoria, Australia; 4 Department of Molecular and Cellular Biology, University of Arizona, Tucson, Arizona, United States of America; 5 Department of Infectious Diseases, St. Jude Children's Research Hospital, Memphis, Tennessee, United States of America; Emory University, United States of America

## Abstract

Relatively little is known about the viral factors contributing to the lethality of the 1918 pandemic, although its unparalleled virulence was likely due in part to the newly discovered PB1-F2 protein. This protein, while unnecessary for replication, increases apoptosis in monocytes, alters viral polymerase activity *in vitro*, enhances inflammation and increases secondary pneumonia *in vivo*. However, the effects the PB1-F2 protein have *in vivo* remain unclear. To address the mechanisms involved, we intranasally infected groups of mice with either influenza A virus PR8 or a genetically engineered virus that expresses the 1918 PB1-F2 protein on a PR8 background, PR8-PB1-F2(1918). Mice inoculated with PR8 had viral concentrations peaking at 72 hours, while those infected with PR8-PB1-F2(1918) reached peak concentrations earlier, 48 hours. Mice given PR8-PB1-F2(1918) also showed a faster decline in viral loads. We fit a mathematical model to these data to estimate parameter values. The model supports a higher viral production rate per cell and a higher infected cell death rate with the PR8-PB1-F2(1918) virus. We discuss the implications these mechanisms have during an infection with a virus expressing a virulent PB1-F2 on the possibility of a pandemic and on the importance of antiviral treatments.

## Author Summary

Influenza A virus is a respiratory pathogen that causes significant morbidity and mortality in infected individuals, particularly during pandemics like the 1918–1919 Spanish Flu pandemic. Recent data suggests that the influenza virus PB1-F2 protein contributes to disease severity. Here, we use data from infected mice together with quantitative analyses to understand how the PB1-F2 protein from the 1918–1919 pandemic strain influences viral kinetics. We find that the rates of virus growth and decay are increased when the 1918 PB1-F2 is present. Our analyses suggest that infection with an influenza virus possessing the 1918 PB1-F2 protein results in a higher rate of viral production from infected cells and a higher rate of infected cell death. These results provide new insights into the mechanisms of PB1-F2 and the virulence and pathogenesis of pandemic strains of influenza.

## Introduction

The most deadly influenza pandemic documented occurred in 1918–1919 with over 40 million deaths worldwide [1]. The strain responsible for this “Spanish Flu” pandemic was believed to have caused significant primary viral pneumonia, although many fatalities are attributed to secondary bacterial infections [2]–[4]. The unparalleled virulence experienced was probably due both to strain novelty and to one or more intrinsic viral properties.

Present in nearly all influenza A virus (IAV) isolates, including highly pathogenic avian strains [5], the newly discovered PB1-F2 protein is believed to have played a role in the extreme virulence of the 1918 pandemic [6]. Found during a search for 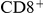 T-cell epitopes, PB1-F2 is a small protein of 87–90 amino acids encoded by an alternate reading frame of the PB1 gene segment [7]. Expression levels of this protein are variable in cells, and it has been found localizing to mitochondria, although it is also present in the cytoplasm and the nucleus [7]–[9]. IAV-induced apoptosis of infected monocytes has been shown to occur with PB1-F2 expression, and is likely due to this protein's ability to target and interfere with mitochondrial functions [7], [8], [10]. The PB1-F2 protein is recognized by the human immune system leading to both humoral and cell-mediated immune responses [7], . Furthermore, PB1-F2 can modulate the type I interferon response in infected cells [13], [14] and result in increased infiltration of monocytes and neutrophils [13]. This was shown to be particularly true for influenza viruses with an amino acid mutation in position N66S in the PB1-F2 protein, which is characteristic of the 1918 strain [13]. Although PB1-F2 is not required for viral replication, it was proposed that the efficiency of replication in epithelial cells may be altered by PB1-F2 interacting with the viral polymerase protein PB1 [15]. This effect, however, has been found to be minor and depend on both cell type and virus strain, although plaque size was significantly larger with a virus that expressed the 1918 PB1-F2 [16].

Using a PB1-F2 knock-out virus, decreased pathogenicity in primary viral pneumonia resulting in rapid viral clearance was demonstrated in a mouse model [17]. It has been found that a single amino acid mutation in PB1-F2 of the 1918 pandemic strain was sufficient to significantly affect the virulence of this virus [13], [18]. However, the effect of the PB1-F2 protein seems to be dependent on both virus and host factors since knock-outs of PB1-F2 on an A/WSN/33 (H1N1) virus (WSN) background did not produce the same effects demonstrated using the A/Puerto Rico/8/34 (H1N1) virus (PR8) [16], [17], [19]. We and others have found that PB1-F2 induces large infiltrates of immune cells [6], [13], [19] and significantly increases the establishment of secondary bacterial pneumonia *in vivo*, whereas PB1-F2 knock-out viruses show decreased pathogenicity [6].

Using genetic information from a 1918 pandemic victim [20], we engineered a virus to express the PB1-F2 protein from the 1918 strain, A/Brevig Mission/1/1918 (H1N1), with a PR8 background [6]. The introduction of this 1918 PB1-F2 created a more deadly virus which resulted in significantly increased viral titers in the first 32 hours compared to its isogenic parent, increased inflammation and increased bacterial establishment and severity [6]. Connections between the mechanisms by which PB1-F2 enhances pathogenicity *in vivo* and the *in vitro* functions, such as cellular apoptosis and polymerase regulation, have recently been investigated [16], [19] but increased inflammation was the most consistent effect of PB1-F2 [16].

To link mechanisms studied *in vitro* with their effects *in vivo*, mathematical models can be used to tease apart the effect of virus replication rates, virus half-life and infected cell life-spans. Recently, several studies have used mathematical formulations to describe influenza virus kinetics in a variety of experimental systems (reviewed in ref. [21], [22]). Target cell limited models have been used in conjunction with nasal wash samples collected from individuals infected with the influenza virus strains A/Hong Kong/123/77 (H1N1) [23] and A/Texas/91 (H1N1) [24], respectively, to estimate important viral kinetic properties [25], [26]. More complicated models have also been developed to investigate the immune responses associated with influenza infection. The adaptive immune response was the focus of one study where mice were infected with H3N2 influenza virus A/Hong Kong/X31 (X31) [27]. A follow-up investigation included a more detailed experimental analysis and inclusion of components of the innate immune response [28]. Another recent model was used to describe an influenza A/equine/Kildare/89 (H3N8) virus infection in Welsh ponies [29] to gain understanding of the contributions of innate immunity and target cell depletion to infection kinetics [30].

A similar set of models have been used to study an influenza infection *in vitro*. These include infecting Madin-Darby canine-kidney (MDCK) cells in a large-scale microcarrier culture with equine influenza virus strain A/equine/Newmarket/1/93 (H3N8) to estimate parameters by fitting a mathematical model to viral measurements taken at various time points [31]. Another study applied these models to viral titer data collected from a hollow-fiber system in which MDCK cells were infected with the influenza A/Albany/1/98 (H3N2) virus [32]. More recently, the kinetics of three influenza viruses, the avian influenza A/Hong Kong/483/97 (H5N1) virus, the seasonal influenza A/New Caledonia/20/99 (H1N1) virus, and the swine-origin influenza A/California/04/09 (H1N1), were modeled and compared using both differential equation and cellular automata approaches with viral titer data from infection of human differentiated bronchial epithelial cells in an air-liquid interface culture [33].

Effectively applying mathematical models to fit data and estimate parameters requires both accurate and frequent measurements of viral loads. The choice of experimental system and the variables measured can influence results. Human nasal wash data provide viral titers over time in a set of individuals but sample only the upper respiratory tract and do not directly assess the lower respiratory tract where severe infections and pneumonia occur. Furthermore, many nasal wash samples contain low titers that cannot be detected, especially early in the infection. On the other hand, *in vitro* samples provide insights into key features of virus production but leave out important components, such as the immune mediated effects, that occur during infection within a host.

To address the influence of the 1918 PB1-F2 on *in vivo* infection characteristics, we infected groups of BALB/c mice with one of two influenza A viruses, A/Puerto Rico/8/34 (H1N1) and a variant expressing the 1918 PB1-F2 protein, and obtained viral measurements from the lungs of individual mice. These data provide information on an infection occurring in the lower respiratory tract. They allow us to compare the kinetics of two virus strains over the course of infection to infer possible mechanisms of PB1-F2 action.

We first analyze these data by comparing viral titers at various times following inoculation. We use linear regression analysis to determine the slopes of viral growth and decay. We then apply a simple model to better quantify the dynamics of infection *in vivo* and understand how the PB1-F2 protein of the 1918 pandemic strain influences kinetics. Using this model, we estimate important infection parameters and evaluate which components, such as virus production or clearance, epithelial cell death, and/or infectivity, are affected by expression of the 1918 PB1-F2. These various analyses suggest which of the processes may be responsible for the effects of PB1-F2 *in vivo*, and we evaluate the relation to previously described mechanisms *in vitro*.

## Results

### Viral Titers of Mice Infected With Influenza PR8 or PR8-PB1-F2(1918)

**Figure 1:**
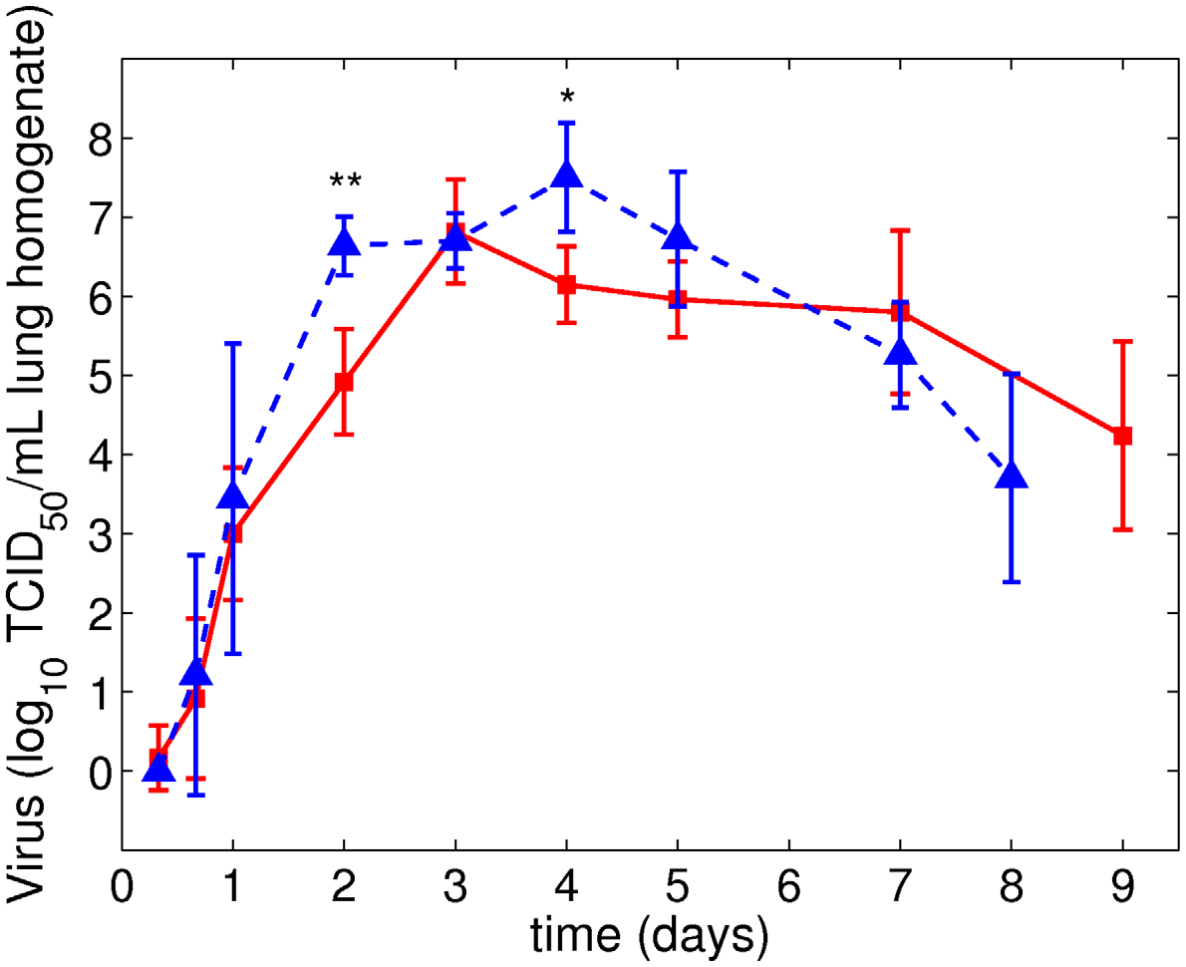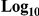 values of viral titers per ml of lung homogenate from groups of 6–10 mice infected intranasally with 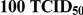 of influenza A virus PR8 (squares) or PR8-PB1-F2(1918) (triangles). Data are given as geometric means 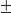 SD. T-tests were used to determine significance of differences of viral titers between these two strains for each time point, 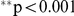, 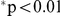.

IAV lung titers, for both PR8 and PR8-PB1-F2(1918), initially increase exponentially reaching peaks up to 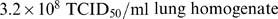. Mice inoculated with PR8 had viral titers peaking at 72 hours postinoculation (p.i.) while mice given the PR8-PB1-F2(1918) virus reached high titers (equivalent to the peak of PR8) earlier at 48 hours p.i. (Figure 1). However, PR8-PB1-F2(1918) titers remain high through 4 days p.i. with peak values around 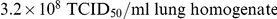. Titer differences at 2 days and 4 days p.i. are statistically significant, 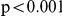 and 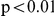, respectively. Viral titers of both strains then decline as the mice recover. All mice survived the experiment.

### Kinetics of Virus Increase and Decline

**Figure 2:**
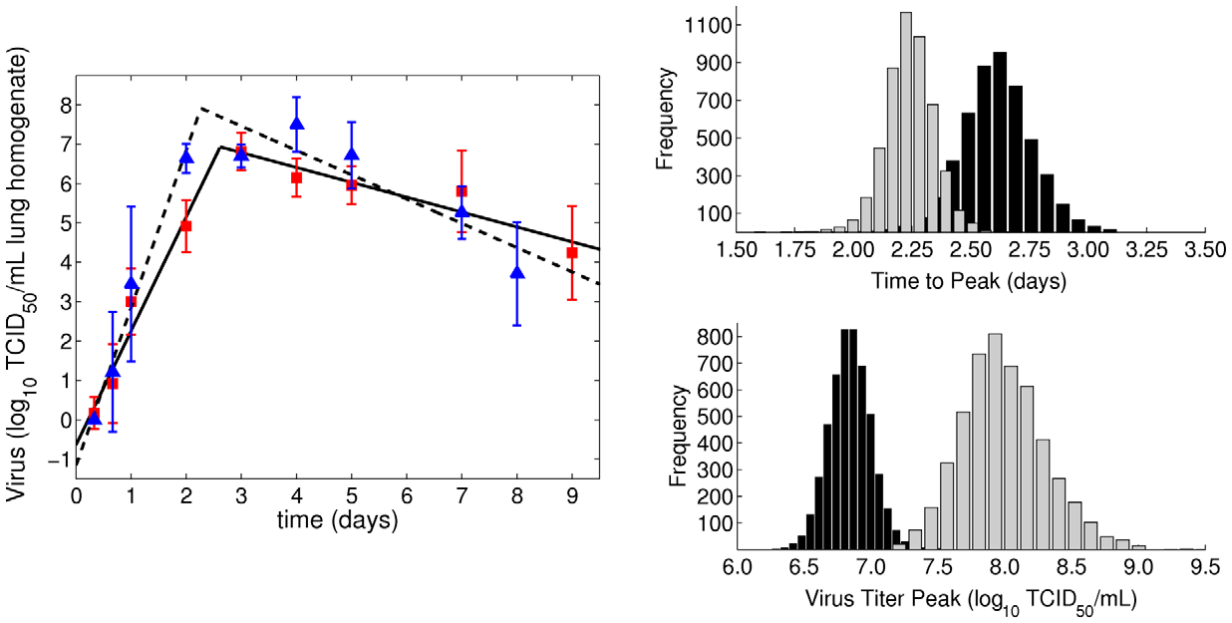
Log-linear fits to lung viral titers in Phases I and II of an influenza infection with PR8 (solid line, squares) or PR8-PB1-F2(1918) (dashed line, triangles). The number of data points included in each phase was determined by finding the two lines that produced the maximum likelihood fit. No data point was allowed to be included in both phases. Distributions of peak times (days) and titers (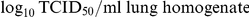), PR8 - black and PR8-PB1-F2(1918) - gray, from bootstrap replicates of the log-linear fits.

Initially, viral titers drop as some virions are cleared while others infect cells that enter an eclipse phase before virus production occurs. After virus production begins, viral titers increase exponentially then reach a peak and subsequently decay exponentially. Because of the striking log-linear structure of the data, we fit two straight lines to the 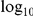 values of viral titers, one to the rise and the other to the decline. By finding the two lines that produce the maximum likelihood fit, the point where the initial rise ends and the decline begins falls between 48 and 72 hours p.i. for both PR8 and PR8-PB1-F2(1918). We define Phase I to be the viral dynamics occurring during the viral load rise, typically the first 0–48 hours, and Phase II as the viral decline, typically 3–9 days p.i. (Figure 2).

We find that Phase I runs from the time of inoculation until 2.6 days p.i. for PR8 and until 2.3 days p.i. for PR8-PB1-F2(1918). Corresponding viral titers at the break points, which can be viewed as imputed peaks, are 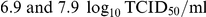, respectively. Using 5000 bootstrap replicates, we calculated the distributions of peak viral titers and the times of these peaks (Figure 2). Using a permutation test [34], we find that both the differences in the peak timing and viral titer value at this peak are statistically significant (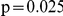 and 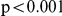, respectively).

In Phase I, the initial intercept value (0 days p.i.) is not significantly different between PR8 and PR8-PB1-F2(1918) (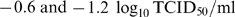, 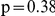, respectively) suggesting the initial inoculum size reaching the lungs is similar for both strains. The slope of PR8-PB1-F2(1918) viral titer increase is higher than that of PR8 by 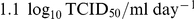 (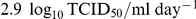 for PR8 versus 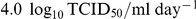 for PR8-PB1-F2(1918), 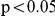). Therefore, we find that viral titers increase more quickly and reach a higher peak value when a PR8 virus containing the 1918 PB1-F2 is given.

In Phase II (3–9 days p.i.), extrapolated viral titers for the two strains at both 3 and 9 days p.i. were not significantly different, 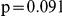 (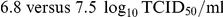 for PR8 and for PR8-PB1-F2(1918), respectively) and 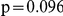 (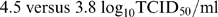 for PR8 and for PR8-PB1-F2(1918), respectively), respectively. Nonetheless, our linear regression analysis of the Phase II viral titer decay, which utilizes all the data between days 3 and 9, suggests that the rate of viral clearance is enhanced in PR8-PB1-F2(1918) infection by 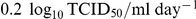 (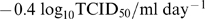 for PR8 versus 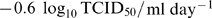 for PR8-PB1-F2(1918), 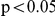). These results are summarized in Table 1 and the best fit lines to PR8 and PR8-PB1-F2(1918) viral titers are shown in Figure 2.

**Table 1:** Slopes, intercepts, peak times and peak values from linear regression analysis of PR8 and PR8-PB1-F2(1918) lung titers.

	Phase I		Peak		Phase II		
	Slope 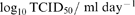	Day 0 Titer 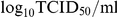	Time days	Titer 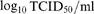	Slope 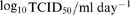	Day 3 Titer 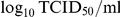	Day 9 Titer 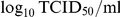
**PR8**	2.89	−0.64	2.62	6.93	−0.38	6.79	4.52
**PR8-PB1-F2(1918)**	4.0	−1.17	2.27	7.91	−0.62	7.46	3.75
p-value	0.032	0.38	0.025	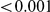	0.044	0.091	0.096

### Estimation of Infection Parameters

#### Infection with PR8

**Figure 3:**
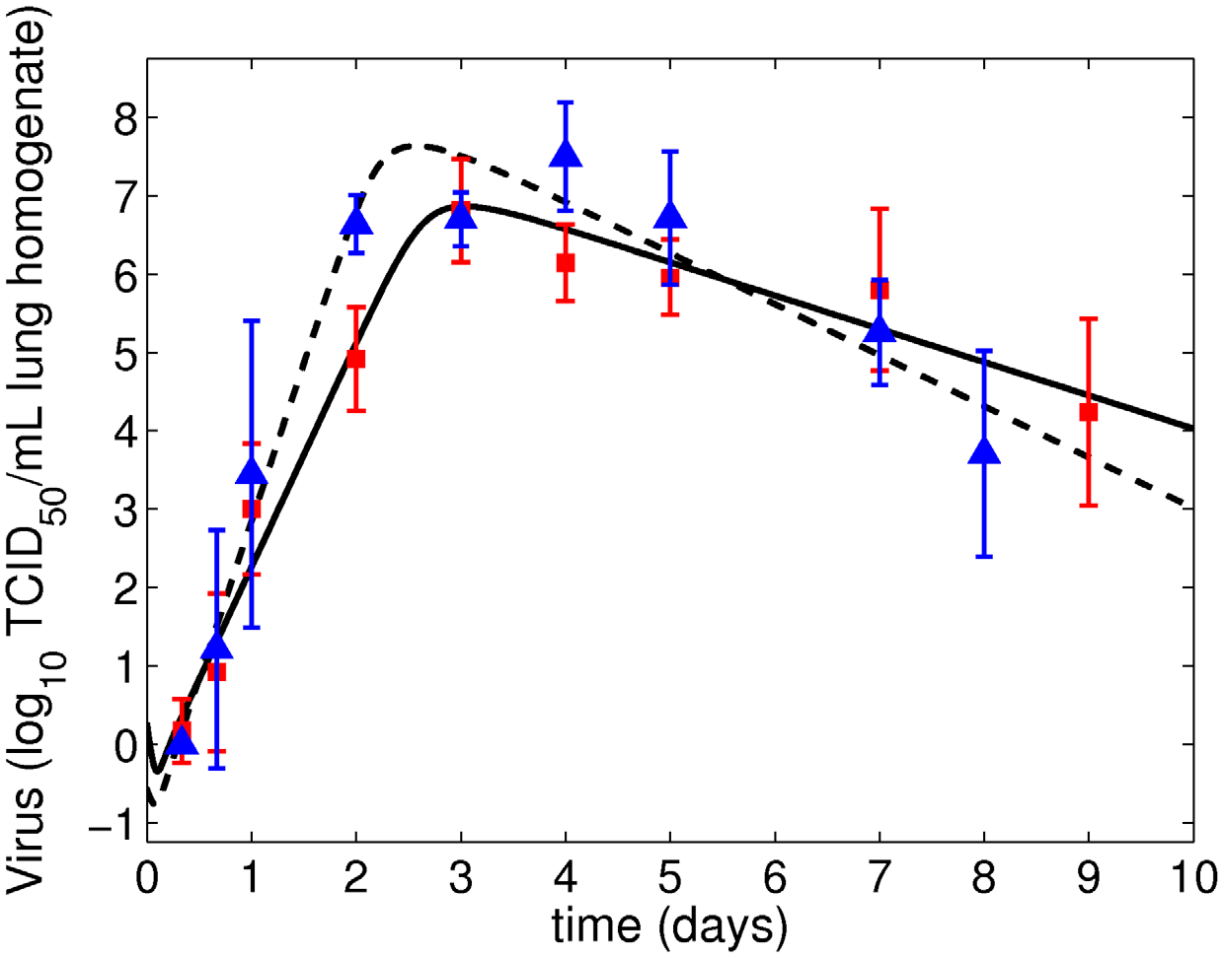
Maximum likelihood fits of the viral kinetic model (**Equations (4)**–**(7)**) to lung titers from individual mice infected with the PR8 virus (solid line, squares) or the PR8-PB1-F2(1918) virus (dashed line, triangles).

We first fit Equations (4)–(7) to PR8 viral lung titers collected over 9 days to estimate model parameters (Table 2). The fit is shown in Figure 3. When the eclipse phase parameter 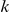 is not fixed, the maximum likelihood estimate lies outside the biologically feasible range, 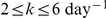 (4–12 hours) [35], [36]. Thus, we fixed 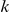 and set it at 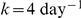 as has been done previously [32], [37], implying that the average eclipse phase length, 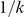, is 6 hours.

**Table 2:** Maximum likelihood estimates of parameter values for influenza infection with PR8 and PR8-PB1-F2(1918).

	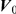 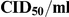	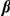 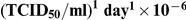	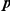 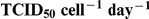	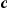 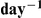	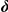 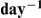
**PR8**	2.0	2.8	25.1	28.4	0.89
95% CI	[0.6 8.4]	[1.3 5.0]	[19.9 58.0]	[14.2 50.0]	[0.6 1.3]
**PR8-PB1-F2(1918)**	0.26	0.91	72.8	9.2	1.5
95% CI	[0.1 1.1]	[0.3 3.4]	[41.3 152.9]	[3.1 50.0]	[0.9 2.4]

For each virus strain, PR8 and PR8-PB1-F2(1918), the MLE initial viral titer (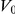), infection rate constant (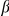), death rate of productively infected cells (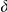), viral release rate per infected cell (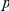), and viral clearance rate (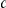). Initial number of target cells (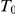) is fixed at 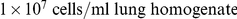, and the transition rate for infected cells to produce virus (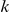) is fixed at 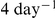.

We also impose a biological consistency condition. If virions are cleared with rate constant 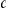, then their average lifetime is 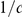. During their lifetime, the average number of cells a virus infects is 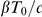. We require that our parameter estimates satisfy 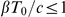 so that, on average, each virion infects at most one cell.

The basic reproductive number, defined as
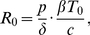
(1)is the product of the average number of virions produced during the lifetime of an infected cell (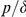) and the average number of cells infected per virion (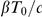) [38]. The parameter estimates for our fits for the infection with PR8 result in 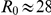. Enforcing the consistency condition produces parameters for which 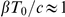 while 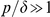 (Table 3). The assays used measure only infectious virus so these parameter values do not reflect the properties of noninfectious virions.

**Table 3:** Infection characteristics for influenza infection with PR8 and PR8-PB1-F2(1918).

	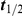 hrs	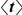 hrs	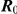	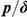	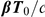
**PR8**	0.6	32.9	27.8	28.2	0.99
95% CI	[0.3 1.2]	[24.5 46.0]	[4.0 340.4]	[15.3 96.7]	[0.3 3.5]
**PR8-PB1-F2(1918)**	1.8	22.3	48.9	49.5	0.99
95% CI	[0.3 5.4]	[16.0 32.7]	[1.0 1863.3]	[17.2 169.9]	[0.1 11.0]

For each virus strain, PR8 and PR8-PB1-F2(1918), the virus half-life (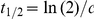), infected cell lifetime (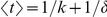), basic reproductive number (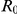), average number of virions produced per infected cell (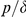), and the average number of cells infected per infectious virion (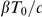) are given (parameter values used are in Table 2). The 95% confidence interval (CI) is given below parameter estimates.

We estimate the half-life (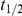) of free infectious PR8 virus to be 0.6 hours. One study found that H3N2 influenza A virions lose infectivity *in vitro* at a rate of 0.105 per hour, which corresponds to a half-life of 6.6 hours [32]. If we assume PR8 loses infectivity at approximately the same rate *in vivo*, then the majority of viral clearance can be attributed to physical removal of viral particles rather than loss of infectivity.

The average time a cell lives while infected with PR8, including both the unproductive and productive stages, is approximately 33 hours. This value is significantly longer than previous estimates of 11.4 hours for infection in the human upper respiratory tract with an H1N1 virus [25] and 19.2 hours for infection *in vitro* with an H3N2 virus [32].

#### Infection with PR8-PB1-F2(1918)

Fitting Equations (4)–(7) to PR8-PB1-F2(1918) viral titers, again fixing 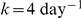 and restricting 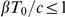, produced parameter estimates different from those for PR8 (Table 2). In particular, for PR8-PB1-F2(1918) we estimate lower values for the infection rate constant (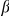), virus clearance rate (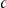) and initial viral concentration (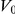), and higher values for the rate of virus production (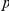) and for the rate of infected cell death (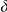).

**Figure 4:**
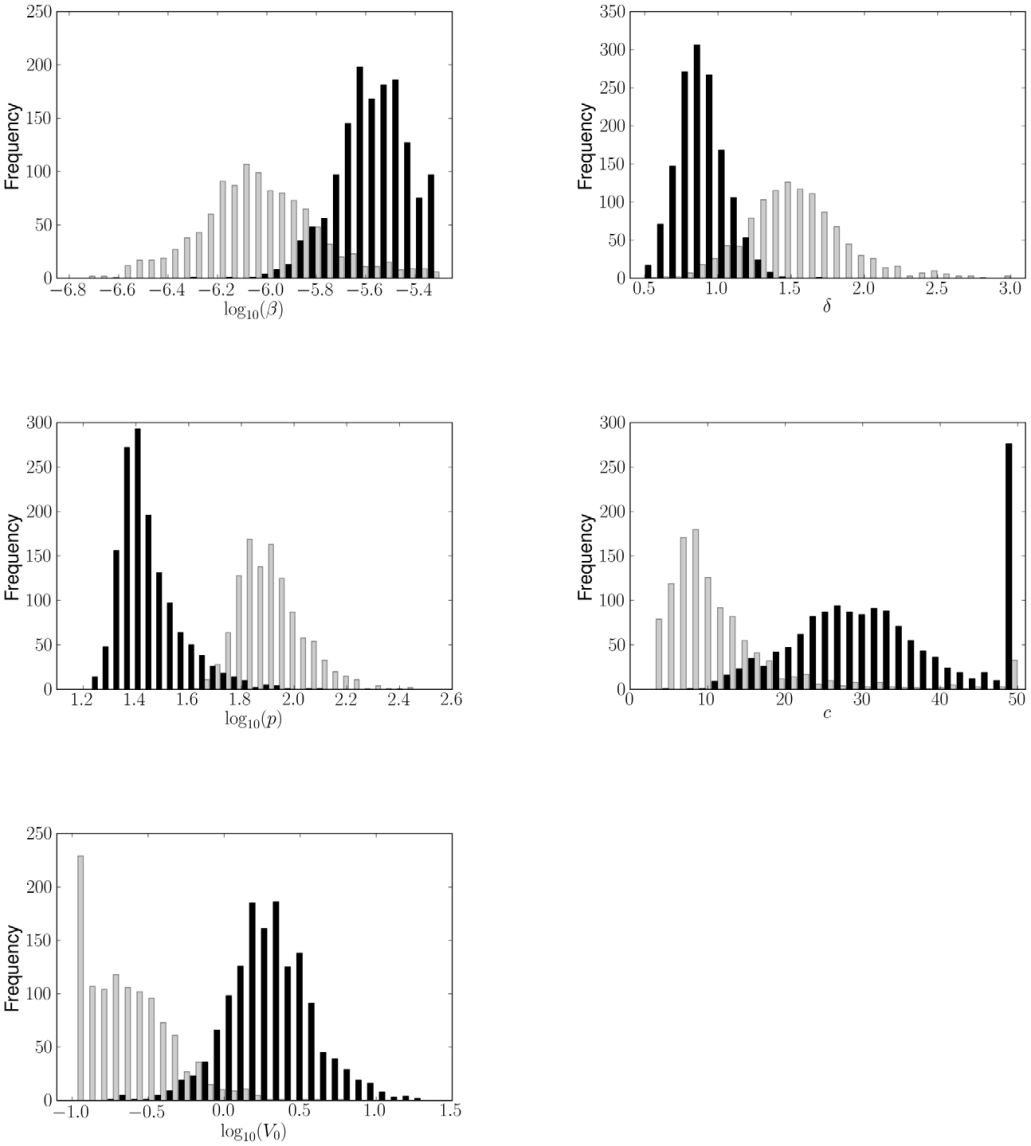
Distributions of the parameter values from bootstrap fits of the viral kinetic model (**Equations (4)**–**(7)**) to lung titers from mice infected with PR8 (black) or PR8-PB1-F2(1918) (gray). Significant differences were detected in the viral production rate (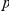, 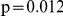), the infected cell death rate (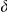, 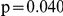) and the initial viral titer (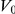, 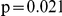).

**Figure 5:**
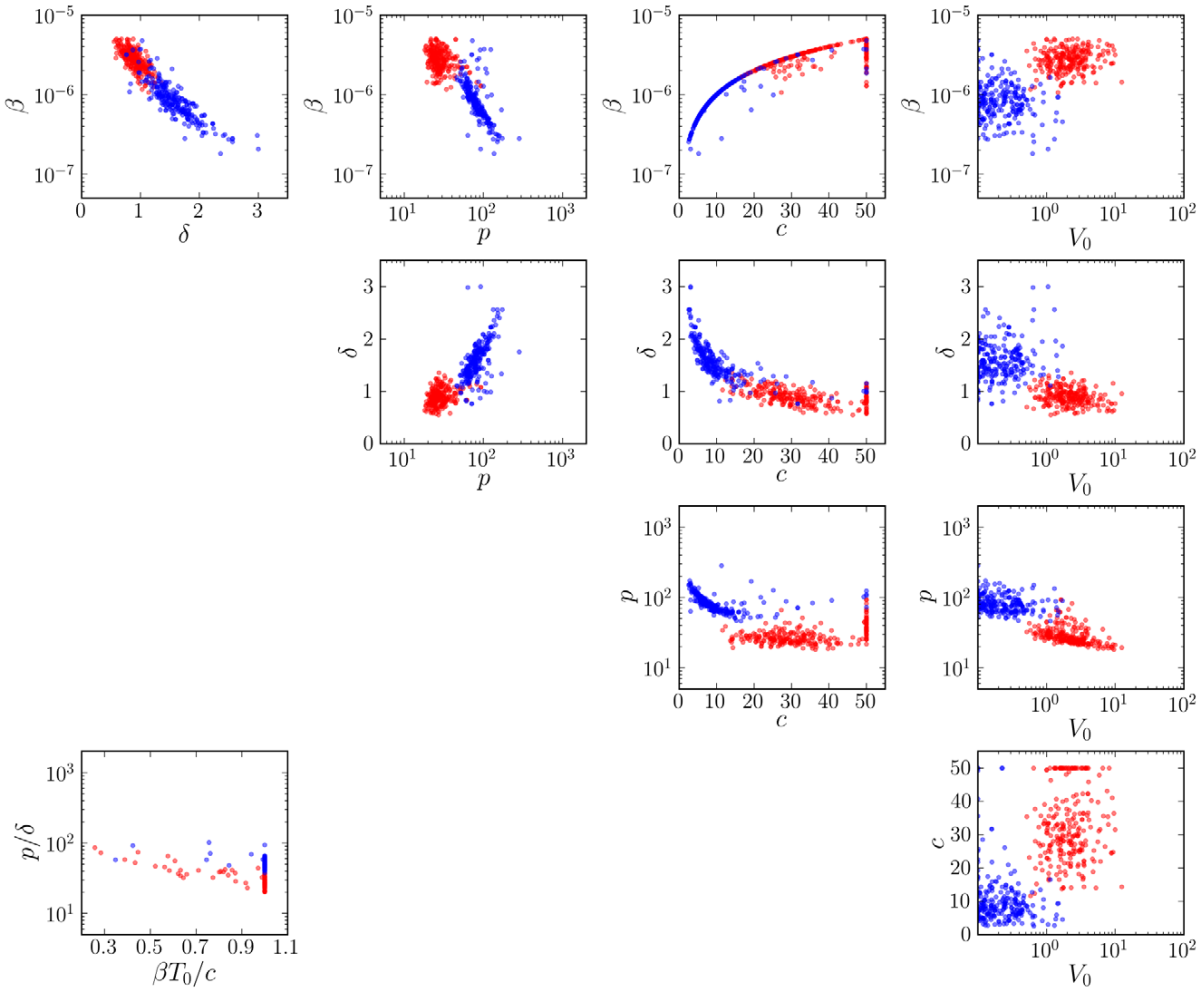
Parameter ensembles from bootstrap fits of the viral kinetic model. Plots of the parameters, in the form of two parameter projections of each fit, and the constraints (bottom left) from bootstrap fits of the viral kinetic model (Equations (4)–(7)) to lung titers from mice infected with PR8 (red) or PR8-PB1-F2(1918) (blue).

Fitting the model to bootstrap replicates of the viral titer data for each strain, the distributions of parameter values were obtained (Figure 4). We find that the differences in the viral production rate (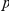), the infected cell death rate (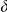) and the initial viral titer (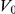) are statistically significant (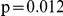, 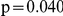, and 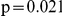, respectively). These differences can be seen in Figure 5, which plots the sets of parameters from fitting the bootstrap data in the form of two-parameter projections (“ensembles”).

Cases in which the two ensembles overlap indicate that the data and the model cannot distinguish those parameters between PR8 and PR8-PB1-F2(1918). These plots demonstrate the strong correlation between the rate of cell infection (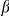) and the rate of viral clearance (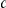) necessary to fit each data set. This correlation is due to the imposed biological consistency condition, 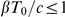. Furthermore, constraining parameter values to lie within predetermined ranges (see Materials and Methods) yielded artificially high bootstrap frequencies at the boundary values for estimates of 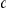 for PR8 and of 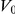 for PR8-PB1-F2(1918) (Figures 4 and 5).

The estimate of the virus clearance rate, 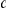, for PR8-PB1-F2(1918) indicates a virus half-life of 1.8 hours, compared to 0.6 hours for PR8, implying that expression of the 1918 PB1-F2 may facilitate virion survival outside of the host cell. The parameter estimates produce an infected cell lifespan of 22 hours for PR8-PB1-F2(1918) versus 33 hours for PR8, suggesting that the 1918 PB1-F2 may act either directly or indirectly to enhance infected cell removal. Furthermore, the values of 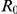 differ between PR8 (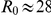) and PR8-PB1-F2(1918) (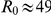). The maximum likelihood parameter estimates and their associated 95% CIs for both PR8 and PR8-PB1-F2(1918) are given in Table 2 and the model fits in Figure 3.

### Connection between Virus Increase and Decline and Infection Parameters

Both the linear regression analysis and the fits of the viral kinetic model to the data show significant differences between virus strains. To link these two analyses, we have investigated how the estimated model parameters relate to the more easily computed linear regression fits to the rising and falling portions of the viral titer curve [39].

We previously derived approximate analytical solutions to Equations (4)–(7) in the increasing (Phase I) and declining (Phase II) portions of the viral kinetic curve [39]. This analysis showed that the virus dynamics after the initial dip in viral titers can be described by
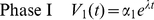
(2)

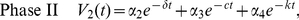
(3)where 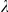 and the 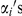 are complex combinations of the parameters (as given in ref. [39]). Using the parameter estimates in Table 2, these expressions give an exponential growth parameter (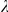) of 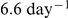 for PR8 and 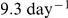 for PR8-PB1-F2(1918), which compare well with the direct estimates from the data (Table 4).

**Table 4:** Link between linear regression analysis and viral kinetic model estimates of the slope and length of viral growth (Phase I) and the slope of virus decay (Phase II).

	Analysis	Initial Viral Titer, 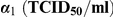	Slope of Exponential Growth, 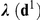	Length of Exponential Growth, 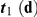	Slope of Exponential Decay, 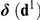
PR8	Approximate Solution/MLE	0.25	6.59	2.14	0.89
	Linear Regression	0.23	6.65	2.62	0.87
				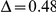	
PR8-PB1-F2(1918)	Approximate Solution/MLE	0.067	9.26	1.80	1.47
	Linear Regression	0.068	9.21	2.27	1.42
				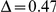	

The MLE parameters in Table 2 were used in the approximate solution [39] of Equations (4)–(7) to find estimates of 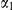, 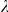, and 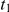. The infected cell death rate (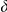) was found to be the slope of exponential virus decay. The intercept value (0 days p.i.) (Table 1) is the effective initial titer and is an estimate of the constant 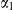 in Equation (2). Values of the slopes found via linear regression (Table 1) were converted from 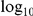 to 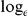 for an accurate comparison.

The approximate solution also provides an estimate of the duration of the exponential growth phase (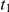) [39]. With the estimated parameters, this phase lasts for 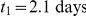 and 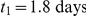 for PR8 and PR8-PB1-F2(1918), respectively. This is approximately 0.5 days less than the peak times found via linear regression, 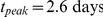 and 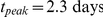, respectively. The difference in these values represents the time between the slowing of exponential growth and the time of the estimated peak, suggesting that the growth of both PR8 and PR8-PB1-F2(1918) starts to slow approximately 12 hours before the viral titer peaks.

In Phase II, 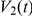 describes virus levels around the peak and the decay throughout infection resolution (Equation (3)) [39]. All three parameters in the exponents, 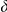, 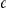 and 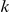, are important in determining the peak shape [39]. However, when the values of these parameters are well separated, the smallest of these three values, 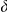 for both PR8 and PR8-PB1-F2(1918) (see Table 2), determines the slope of virus decay (as demonstrated in [39]). This allows us to use the slope of the linear regression as an estimate of the infected cell death rate. The value of 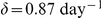 and 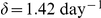 for PR8 and PR8-PB1-F2(1918), respectively, from the linear regression lie within the 95% confidence intervals of the maximum likelihood estimated (MLE) values of 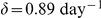 and 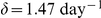.

## Discussion

We explored the *in vivo* kinetics of the mouse adapted PR8 and a variant of PR8 that expresses the PB1-F2 protein from the 1918 influenza strain (PR8-PB1-F2(1918)) using both experimental and mathematical models. The 1918 PB1-F2 protein differs by only 8 amino acids from that in the PR8 strain [6]. Since it may only require a single amino acid mutation in PB1-F2 to influence pathogenicity [13], [18], PR8 and PR8-PB1-F2(1918) may differ significantly in virulence. Furthermore, the 1918 PB1-F2 protein is thought to have a direct role as a virulence factor during both primary viral and secondary bacterial pneumonia [6].

Influenza viral titers in the lungs of the mice over the course of an acute infection exhibit exponential growth for 2–3 days, then briefly level out near the peak, and finally decline exponentially. Lung titers in mice infected with each strain exhibit somewhat distinct patterns of growth. The frequent sampling of these data showed that the PR8-PB1-F2(1918) virus reaches significantly higher titers as soon as 48 hours into the infection, with titers remaining elevated for 2 days before finally declining. In contrast, PR8 viral titers grow more slowly, reaching a peak at 72 hours p.i.. This rapid spread may indicate a potential for larger amounts of viral shedding early on and lead to an increased probability of effective transmission.

We also calculated the basic reproductive number, 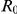, which is the average number of cells one infected cell will infect over its lifetime when placed in a population of cells fully susceptible to infection. 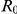 can be calculated from Equation (1). Our estimate of the value of 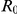 for an infection with PR8 is large, 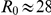, but the value for an infection with PR8-PB1-F2(1918) is even larger (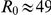). Both of these values are comparable to those estimated from fitting the viral kinetic model to human nasal wash samples, where 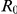 estimates ranged from 3.5 to 75 [25].

Quantifying the differences between infections with PR8 and PR8-PB1-F2(1918) by fitting straight lines to what we term Phase I (initial virus growth) and Phase II (virus decay from the peak) of the 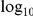 viral titer kinetics confirmed the higher rate of increase and higher rate of decline of the PR8-PB1-F2(1918) virus and the existence of an earlier and higher peak. Our analyses suggest that viral growth generally slows 12 hours before a peak is reached even when virus peaks are distinct, as was the case for PR8 and PR8-PB1-F2(1918). The higher growth rate of PR8-PB1-F2(1918) suggests that virus may either have a higher rate of cell infection (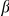) or a higher rate of viral production (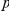) per infected cell. However, using approximate solutions of the mathematical model [39] in combination with the results from our model fits, we determine that the increased growth rate of PR8-PB1-F2(1918) is likely a consequence of increased viral production.

The more rapid decline of PR8-PB1-F2(1918) could be due to more rapid death of infected cells (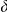). More rapid clearance of virus (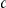) generally does not have a substantial effect on the net rate of virus decline after the peak [39]. Despite the differences in viral growth and decay rates, the lung viral titers shortly after the estimated peak (3 days p.i.) and those near the end of infection are similar between PR8 and PR8-PB1-F2(1918). Therefore, each infection is cleared in roughly the same length of time.

Fitting the viral kinetic model (Equations (4)–(7)) to estimate infection parameters allowed us to examine the differences in viral kinetics due to the insertion of the 1918 PB1-F2 into PR8 in more detail. Two parameters, the rate of viral production (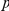) and the rate of infected cell death (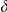), emerged as the leading candidates for the processes affected most by the presence of the 1918 PB1-F2. However, there was some indication that 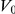, the amount of virus initially reaching the lungs, is different between the two viruses. We do not have a reason to believe that insertion of the 1918 PB1-F2 into PR8 would directly influence 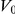, and the difference noted could be due to the simplified nature of the model. However, PB1-F2 does cause inflammatory changes in the lung [19], which in turn could affect viral distribution. Whether this effect would occur early enough in the infection to correspond to a change in 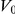 is unclear, but PB1-F2 is produced within the first 6 hours of infection [16]. Furthermore, the clustering of 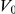 at the lower bound for PR8-PB1-F2(1918) is surprising and suggests that early data is inadequate to precisely estimate 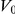.

For a wild-type PR8 infection, the viral kinetic model yielded an estimate of the free virus half-life (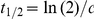) of 0.6 hours, while 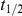 for PR8-PB1-F2(1918) was 1.8 hours. With large 95% CIs, we could not detect a statistically significant difference between the estimated values of the viral clearance rates (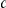). However, these differences could indicate that expression of a virulent PB1-F2, possibly corresponding to the N66S mutation [13], [18], decreases the clearance ability of the immune system. Whether this is due to death of phagocytic cells, an effect on mucocilliary clearance, or an effect on the possible decrease in innate defenses caused by the 1918 PB1-F2 [13] cannot be discerned from our modeling efforts. A competent immune system is essential to clear virus as immunocompromised individuals can exhibit persistent viral shedding [40]. PB1-F2 has been shown to sensitize monocytes to proapoptotic stimuli [7], [8], [10], which could explain an extended survival of PR8-PB1-F2(1918) outside of a cell. Long lasting free infectious virus may have a greater chance to infect cells, create a more severe infection and increase the likelihood of transmission between hosts.

We note that the balance of a higher death rate of infected cells and a larger production of virus leads to only minor differences in the viral titers of these two infections [19], which we were able to detect only due to the enhanced sampling of viral titers and the kinetic modeling of the data. We have to interpret these results of our modeling analyses in the context of our recent studies to understand mechanistically the effect of PB1-F2, particularly from the 1918 strain.

The influence PB1-F2 has on viral polymerase function [15], although modest [16], could at least partially explain the higher estimate of the per cell production, 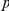, for the PR8-PB1-F2(1918) virus. In a plaque forming assay, plaques generated by PR8-PB1-F2(1918) virus were significantly larger than those generated by PR8 [16], which would be consistent with a higher per cell production rate. Since plaque assays only measure infectious virus, an effect of PB1-F2 on the polymerase that increases the fraction of infectious virions produced or accelerates viral production would correspond to an increase in the infectious virus production rate, 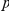. However, this increased production could be due a delayed innate immune response resulting from the ability of the 1918 PB1-F2 to inhibit the type I interferon response in infected cells [13].

The 1918 PB1-F2 also seems to decrease the average survival time of a productively infected cell (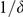) from 27 hours for PR8 to 16 hours for PR8-PB1-F2(1918). Whether this is a host effect or a virus effect is not known. Induction of cell death by PB1-F2 has been shown to be strain-specific and not significantly increased early in the infection with the 1918 PB1-F2 [19]. Since PB1-F2 may have maximum production 6–8 hours p.i. [16], the differences in increased cell death we found may be an indirect effect that occurs downstream. The associated cell damage may then contribute to the immune cell infiltration that has been found [6], [13], [19], however a direct link has yet to be established [19]. The mechanism of cell death, whether directly caused by PB1-F2 within the cell or by an increased immune system response, remains unclear.

Interestingly, in cell culture, replication of PR8 and PR8-PB1-F2(1918) is similar [6]. This could be due to the combination of effects our modeling has revealed. Cells infected with PR8-PB1-F2(1918) could produce virus at a higher rate than cells infected with PR8, but if such cells also lived a shorter time, the amount of virus sampled *in vitro* at each time point could be similar for the two viruses.

The immunopathology associated with the PB1-F2 protein has recently been investigated and is thought to play a role during influenza pathogenesis [19]. Enhanced inflammation in the lungs, at least partially from an immune response dominated by macrophage and neutrophil infiltration, has been shown [6], [13], [19]. The mechanisms of immunopathology are unknown but some studies suggest that PB1-F2 regulates pathways involved in the innate immune response, particularly the activation of type I interferon pathway genes [13], [14]. The model we use does not include specific host responses and is unable to address the mechanisms involved in the increased inflammatory response. A more complicated model involving components of the immune system and quantitative measurements of these cells and cytokines would be necessary and is a focus of future work.

The viral kinetic model we use has previously been applied to data collected from nasal wash samples in humans [25], in cell culture [32] and now from murine lung samples. Parameter estimates found in these studies differ (discussed in detail in [22]). Here, when we imposed the condition that each virus, on average, infects at most one cell such that 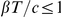, we found that the average number of cells infected per infectious particle is close to one at the initiation of infection when 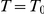 and target cells are most abundant. This suggests that once virus gets into the lung, target cells are sufficiently plentiful that almost every infectious virion finds a target cell to infect before being cleared.

The burst size from an influenza infected cell has been estimated to be 18,755 virions (for infection of MDCK cells with an equine influenza virus (H3N8)) [31], where approximately 1 in 100–500 virions are infectious [41], [42]. If these values are accurate for influenza viruses in general and we assume that 1 in 500 virions is infectious, our findings that the average number of infectious virions produced per cell (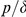) is 28 for PR8 and 49 for PR8-PB1-F2(1918) suggest that the burst size for PR8 is 14,000 virions and for PR8-PB1-F2(1918) is 24,500 virions.

Several variants of the model have been explored by us (results not included) and others [32] to remedy possible violation of the condition 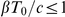. One model included a term for the loss of free virus from infecting cells, such that the equation for free virus becomes 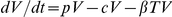. Including this term ensures that the average number of cells infected per virion at the initiation of infection (now 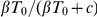) is less than one, but the estimated values of 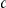 became unrealistically large such that 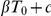 in the denominator once again became approximately equal to 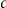. While this attempt was unsuccessful overall, creating a more accurate model formulation remains a focus of future work.

Here, we have shown how the 1918 PB1-F2 protein can have significant effects on infection dynamics. Mathematical models can be utilized to predict the behavior of this virus relative to PR8 and link the biochemical, cellular, immunological, and population levels. Furthermore, the equations describing viral growth and decay (Equations (2)–(3)) can be easily used with results obtained from the linear regressions providing a simple approach to investigate certain aspects of infection without estimating parameters. Fully understanding the effects PB1-F2 has in various host and virus contexts is crucial to successfully prepare for circulation of a strain that may be only a single amino acid mutation away from having pandemic potential.

## Materials and Methods

### Ethics Statement

All experimental procedures were approved by the Animal Care and Use Committee at SJCRH under relevant institutional and American Veterinary Medical Association guidelines and were performed in a Biosafety level 2 facility that is accredited by AALAAS.

### Mice

Adult (6–8 wk old) female BALB/cJ mice were obtained from Jackson Laboratories (Bar Harbor, ME). Mice were housed in groups of 4–6 mice in high-temperature 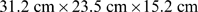 polycarbonate cages with isolator lids. Rooms used for housing mice were maintained on a 12∶12-hour light∶dark cycle at 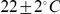 with a humidity of 50% in the biosafety level 2 facility at St. Jude Children's Research Hospital (Memphis, TN). Prior to inclusion in the experiments, mice were allowed at least 7 days to acclimate to the animal facility. Laboratory Autoclavable Rodent Diet (PMI Nutrition International, St. Louis, MO) and autoclaved water were available ad libitum. All experiments were performed in accordance with the guidelines set forth by the Animal Care and Use Committee at St. Jude Children's Research Hospital.

### Influenza Viruses

Viruses used in the experimental model consist of (i) the mouse adapted influenza A/Puerto Rico/8/34 (H1N1) (PR8), and (ii) a genetically engineered influenza virus referred to as “PR8-PB1-F2(1918).” The latter virus, generated at St. Jude Children's Research Hospital, has a PR8 backbone with an eight amino acid change in the PB1-F2 protein to match the protein from influenza A/Brevig Mission/1/1918 (H1N1). For details on the construction of this virus, see McAuley et al. (2007).

### Infection Model

The dose infectious for 50% of tissue culture wells (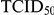) was determined by interpolation using the method of Reed and Muench [43] using serial dilutions of virus on Madin-Darby canine kidney (MDCK) cells. For infection experiments, virus was diluted in sterile PBS and administered at a dose of 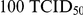 intranasally to groups of 6–10 mice lightly anesthetized with 2.5% inhaled isoflurane (Baxter, Deerfield, IL) in a total volume of 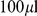 (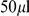 per nostril). Viral measurements were obtained from samples of individual lung homogenates at 8, 16, 24, 48, 72 hours p.i. and 4, 5, 7, 8 (PR8-PB1-F2(1918) only), 9 (PR8 only) days p.i.. Mice were weighed at the onset of infection and each subsequent day for illness and mortality.

### Lung Titers

Mice were euthanized by CO2 asphyxiation. Lungs were aseptically harvested, washed three times in PBS, and placed in 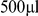 sterile PBS. Lungs were mechanically homogenized using the Ultra-Turrax T8 homogenizer (IKA-werke, Staufen, Germany). Lung homogenates were pelleted at 10,000 rpm for 5 minutes and the supernatants were used to determine the viral titer for each set of lungs using serial dilutions on MDCK monolayers.

### Linear Regression

We used the statistical programming language R [44] to perform linear regression of the 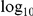 values of viral titer during the initial rise in viral titers and during the viral decay following the peak. To determine the appropriate subset of data to include in each of these phases, we used a maximum likelihood method to find the optimum break point. We did not allow any data point to be included in both phases.

### Mathematical Model

We consider a target cell limited model that incorporates an eclipse phase, originally presented in Baccam et al. (2006), to describe IAV kinetics. We chose this model to analyze the viral titer data because of its simplicity and its proven ability to estimate parameters from viral titer data obtained from both human nasal wash [25] and cell culture [32] infection systems. This model depicts an influenza infection using four populations: susceptible epithelial (target) cells (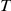), two sets of infected cells (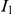 and 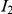), and free virus (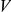). Target cells become infected at a rate 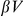 per day. Newly infected cells (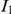) enter an eclipse phase before virion production begins. This period tends to be rather short, e.g., 4–6 hours, and for simplicity we assume no cell death occurs during this period. Cells, 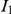, transition to productively infected cells (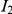) at a rate 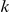 per day. Productively infected cells are lost (e.g., by apoptosis, by viral cytopathic effects or by removal by immune cells) at a rate 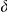 per day. The average total infected cell lifetime is 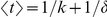. Virus production occurs at a rate 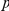 per cell per day, and virions are cleared at a rate 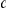 per day (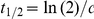 is the virus half-life). The following equations represent these dynamics.
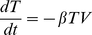
(4)

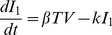
(5)

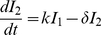
(6)

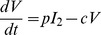
(7)Data and models represent only infectious virus. Noninfectious virus is not detected by the experimental assay used and is not included in the model. We note that this model does not specify mechanisms for a given process. For example, 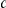 and 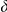 encompass viral effects and different immune mechanisms. Thus, it is possible that some of the parameters change with time. Here, we assume that all parameters are constant and explore how well this model fits the observed viral titer data.

### Parameter Estimation

The curve-fitting method we use is a maximum likelihood estimation (MLE) routine written in Matlab. We assume errors of the 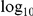 titer values are normally distributed. The negative log-likelihood is minimized across parameter regimes using the Matlab minimization subroutine (*fmincon*) and ODE solver (*ode45*) to compare experimental and predicted values of 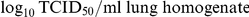. Fit quality is determined by the log-likelihood (LL) value.

To more fully explore and visualize the regions of parameter space consistent with the model and data for each strain and to ensure that the minimum found by the MLE routine was a global rather than a local minima, we use a second method that fits the model to 1000 bootstrap replicates of each data set [34]. For each bootstrap data set, ten fits are run starting from the overall best-fit parameters and perturbing each parameter uniformly within 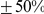. A bootstrap fit was considered successful if the three best log-likelihood fits yielded values within 0.05. For each best-fit estimate, we provide a 95% confidence interval (CI) computed from the bootstrap replicates [34]. These calculations were performed with the software package SloppyCell [45], [46].

In our fits, we placed bounds on the parameters to constrain them to physically realistic values. Since biological estimates are not available for all parameters, we specified ranges that are reasonably large based on previous estimates (reviewed in Ref. [22]). We allowed the rate of infection, 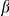, to vary between 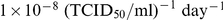 and 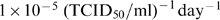, and the rate of viral production, 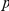, to vary between 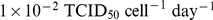 and 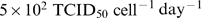. The rate of infected cell death, 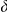, was given a lower limit of 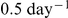, which corresponds to an average infected cell lifespan of 48 hours, and an upper limit of 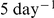, which corresponds to an average infected cell lifespan of 4.8 hours. We set the upper bound on the viral half-life (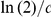) to be 8 hours (i.e., 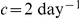) and the lower bound to be 20 minutes (i.e., 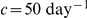). Previous estimates of 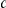 for influenza infection in mice resulted in 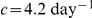 [28], and estimates for other viruses, such as HIV (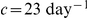 [47]) and hepatitis C virus (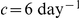 [48]), are in the given range. For the initial viral concentration, 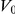, a lower limit of 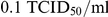 was imposed. Given that a typical lung homogenate is 1–1.5 ml (observed from our experiments), we chose this value since at least one infectious virion is required to initiate an infection and by naively assuming that one or a few infectious virions correspond to a 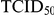. The upper limit on 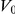 was set to 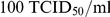.

In each fit, the initial number of target cells, 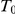, is fixed. Stafford et al. (2000) [49], in the context of an HIV model, showed that it is not possible to estimate both the rate of virus production, 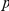, and the initial number of target cells, 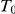, as the model solution involves only the product 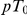. A similar calculation can be done using Equations (4)–(7); therefore, we have chosen to fix 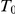 and let the viral production parameter 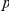 vary in our estimations. We fixed 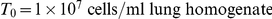 based on an estimate of the total number of type I and type II epithelial cells in the alveolar region of the murine lung [50], and the fact that the total volume of lung homogenate is about 1 ml. The initial number of target cells needs to be given as a density so that units in the model are consistent. By fixing 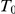 at this value, we are assuming that not all lung epithelial cells are targets for infection. It is possible that some cells are not targets, say due to lack of access of the virus or due to innate immune responses [30].
